# Competition Reduces Response Times in Multiparty Conversation

**DOI:** 10.3389/fpsyg.2021.693124

**Published:** 2021-09-16

**Authors:** Judith Holler, Phillip M. Alday, Caitlin Decuyper, Mareike Geiger, Kobin H. Kendrick, Antje S. Meyer

**Affiliations:** ^1^Max Planck Institute for Psycholinguistics, Nijmegen, Netherlands; ^2^Donders Institute for Brain, Cognition, and Behavior, Radboud University, Nijmegen, Netherlands; ^3^University of York, York, United Kingdom

**Keywords:** multi-party conversation, turn-taking, turn transitions, competition, response times, response latencies

## Abstract

Natural conversations are characterized by short transition times between turns. This holds in particular for multi-party conversations. The short turn transitions in everyday conversations contrast sharply with the much longer speech onset latencies observed in laboratory studies where speakers respond to spoken utterances. There are many factors that facilitate speech production in conversational compared to laboratory settings. Here we highlight one of them, the impact of competition for turns. In multi-party conversations, speakers often compete for turns. In quantitative corpus analyses of multi-party conversation, the fastest response determines the recorded turn transition time. In contrast, in dyadic conversations such competition for turns is much less likely to arise, and in laboratory experiments with individual participants it does not arise at all. Therefore, all responses tend to be recorded. Thus, competition for turns may reduce the recorded mean turn transition times in multi-party conversations for a simple statistical reason: slow responses are not included in the means. We report two studies illustrating this point. We first report the results of simulations showing how much the response times in a laboratory experiment would be reduced if, for each trial, instead of recording all responses, only the fastest responses of several participants responding independently on the trial were recorded. We then present results from a quantitative corpus analysis comparing turn transition times in dyadic and triadic conversations. There was no significant group size effect in question-response transition times, where the present speaker often selects the next one, thus reducing competition between speakers. But, as predicted, triads showed shorter turn transition times than dyads for the remaining turn transitions, where competition for the floor was more likely to arise. Together, these data show that turn transition times in conversation should be interpreted in the context of group size, turn transition type, and social setting.

## Introduction

In everyday conversation, speakers’ turns are well coordinated in time. As noted in a seminal article by Sacks et al. (1974), turns alternate such that most of the time only one person talks, and the gaps and overlaps between their turns are short. For instance, for a corpus of polar (yes/no) questions in ten languages Stivers et al. (2009) found a mean turn transition duration of around 200 ms when averaging across languages and little variation between languages. For a corpus of Dutch, English, and Swedish conversations Heldner and Edlund (2010) reported similar values. An important issue in current psycholinguistic work on conversation is how interlocutors achieve this tight coordination of their turns. The short turn transition times are remarkable because they contrast sharply with the much longer speech onset latencies observed when participants in psycholinguistic experiments produce simple utterances. For instance, naming an object takes at least 600 ms (e.g., Indefrey and Levelt, 2004) and planning a sentence describing a scene can easily take more than a second (Griffin and Bock, 2000; Konopka, 2012). How can speakers be so fast to respond to each other in conversation when it takes them so long to plan simple utterances in the laboratory? As we review below, many variables have already been identified that likely support fast turn-taking in natural conversation. In the present paper, we explore the impact of a variable that has not received much attention in the literature: competition for turns, which, we argue, shortens turn transition times in multi-party conversations compared to dyadic conversations. It may also contribute to explaining the discrepancy between long response times in laboratory studies of speech planning and short turn transition times in corpora of conversational speech.

Levinson and Torreira (2015; see also Levinson, 2016) proposed that speakers achieve fast transitions between their turns by being proactive: They do not await the end of the partner’s turn, but begin to plan a response as soon as possible. For example, a guest in a restaurant hearing the waiter say “Can I.?” may often be able to guess the continuation of the question (“…get you anything else?”) or at least its gist, and plan a response after the first couple of words. This Early Planning Hypothesis plays a central role in Levinson and Torreira’s model of turn taking. Interlocutors begin to plan a response to their partner as soon as they have sufficient information to do so and launch it when the end of the turn is imminent.

Several laboratory studies have examined whether speakers indeed already begin to plan an utterance while still listening to their partner. This is not self-evident because both comprehending and planning utterances require cognitive resources, and because the conceptual and linguistic encoding processes may interfere with each other (e.g., Ferreira and Pashler, 2002; Cleland et al., 2012; Boiteau et al., 2014). In these studies, participants heard utterances and had to respond as quickly as possible. The critical manipulation was that the cue to the answer appeared either early or late in the utterance. If, as the Early Planning Hypothesis predicts, utterance planning begins as soon as possible, the response times should be shorter when the cue appears early than when it appears late in the utterance. As an example, in the first study of this type, Bögels et al. (2015) used quiz questions, where the cue to the answer appeared early, as in “Which character, also called 007, appeared in the famous movies?” or late, as in “Which character from the famous movies is also called 007?” They found that the participants responded faster, on average by about 300 ms, in the former than in the latter condition (means: 640 versus 950 ms). This suggests that they must have begun planning their utterances earlier in the early than in the late cue condition, as predicted by Levinson and Torreira’s Early Planning Hypothesis. Later studies using similar paradigms provided further strong support for this hypothesis (Barthel et al., 2016; Bögels et al., 2018; Corps et al., 2018, 2019; Meyer et al., 2018).

Though these studies unambiguously supported the Early Planning Hypothesis, they consistently failed to elicit latencies that were as short as the mean turn transitions obtained in quantitative analyses of conversational corpora. These are, as noted above, often around 200 ms. For instance, in the study by Bögels et al. (2015), the mean response time in the early cue condition was 640 ms; in the study by Barthel et al. (2016) it was 806 ms; and in the study by Corps et al. (2018, Experiment 2b) it was 484 ms. This discrepancy indicates that early planning by itself does not suffice to explain the speed of conversational turn-taking and that there must be other factors at play that are absent in typical laboratory experiments.

A number of potentially important factors have been discussed in the literature. For instance, in natural conversation speakers can often refer to mutually shared knowledge (also called “common ground”), which facilitates understanding and the generation of appropriate responses (Clark, 1996; Brown-Schmidt et al., 2015). Relatedly, interlocutors may converge in their use of words and phrases. Over the course of an interaction, this may lead to increasing alignment of their conceptual and linguistic representations, which facilitates mutual understanding and responding (e.g., Pickering and Garrod, 2004; Garrod and Pickering, 2009; Branigan and Pickering, 2017; but see Healey et al., 2014). Such alignment can also arise in laboratory contexts (e.g., Garrod and Anderson, 1987; Brennan and Clark, 1996; Branigan et al., 2000). However, often the semantic and/or syntactic structure of the utterances to be produced in a laboratory study is largely predetermined, preventing spontaneous facilitatory alignment (e.g., Sjerps and Meyer, 2015; Barthel et al., 2017). Furthermore, conversation often features very short contributions (“yeah”) and utterances beginning with turn-initial particles, which are likely to be fast to initiate, but do not appear in typical laboratory speech (e.g., Knudsen et al., 2020). Finally, natural conversations are often multimodal (e.g., including manual, head and facial gestures in addition to speech) and the presence of visual information may substantially facilitate the comprehension and production of utterances (Holler et al., 2018). In contrast, most psycholinguistic experiments assessing the Early Planning Hypothesis have allowed the use of spoken language only.

In short, there are many potential reasons why laboratory-recorded response times are much longer than turn transition times in conversation. In this paper, we draw attention to a factor that seems to have been largely overlooked so far: competition for turns, which may arise in multi-speaker conversations but is much less likely to occur in dyadic conversations and is absent from standard laboratory experiments where participants are tested individually.

To elaborate, in a standard psycholinguistic experiment, participants are tested individually and all of their responses (perhaps with the exclusion of errors and some outliers, i.e., responses with extremely short or long latencies) are recorded and averaged. The same is likely to apply in analyses of dyadic conversations. Here participants abide by common turn-taking rules, which involves that the current speaker gives the other speaker(s) the opportunity to take the turn before continuing to speak themselves (Sacks et al., 1974). This means that in quantitative corpus analyses of dyadic conversations all speakers’ planned responses (except perhaps for some responses with very pronounced delays foreshadowing dispreferred responses; see Kendrick and Holler, 2017) tend to be realized and recorded.

The situation is different in multi-party conversations. Here several speakers may wish to speak but typically only one person, the fastest responder, obtains the next turn (Sacks et al., 1974). Thus, those turns that may have been planned but were never issued because someone else responded faster will not enter into the analysis. Alternatively, two or more speakers may start to speak almost at the same time, but in a quantitative corpus analysis the turn transition time would typically still be the fastest responder’s time, or such cases would be removed from the analysis altogether. In short, in multi-party conversation, only the fastest of the competing responses are included in the mean turn transition time, whereas the mean for dyadic conversations is based on almost all responses.

To illustrate the impact of this sampling bias on typical response times we describe the results of simulations showing how the mean and median response times from a laboratory experiment would change, relative to the mean and median of all response times, if for each trial only the fastest response times from two or more participants were recorded and averaged. For instance, if participant A had a response time of 650 ms and participant B had a response time of 700 ms, either both latencies or only A’s latency would be entered into the dataset. These simulations show the impact of recording all or a subset of the response times and combining them in a mean or median. They do not concern the ways the speakers’ behavior may change when they talk in smaller or larger groups.

Then we turn to actual conversations. As discussed above, in quantitative analyses of multi-party conversation, the recorded turn transition time is the response time of the fastest speaker. It follows that as more people participate in a conversation, the mean turn transition time, which is the mean fastest response time, should decrease even when the individual speakers’ behavior is not affected by the number of participants. The same holds for the median. Of course, the speakers’ behavior may change as well. For instance, speakers may begin to plan or launch utterances earlier as they compete for the floor with more co-participants. Although conversation as a whole may be characterized as collaborative, there is often a competitive element to turn-taking (French and Local, 1983; Schegloff, 2000; Kurtić et al., 2013), arising perhaps most clearly in multi-party conversation (e.g., Sacks et al., 1974, p. 712).

No systematic quantitative comparison of turn transitions in dyadic and multi-party conversations appears to exist to date. The existing quantitative studies of turn transition times focused on dyads (e.g., ten Bosch et al., 2004; Heldner and Edlund, 2010; Roberts et al., 2015) or on multi-party conversations (Girard-Groeber, 2015; Holler et al., 2018) or did not distinguish between dyadic and multi-party conversations in their analyses (e.g., Stivers et al., 2009; de Vos et al., 2015). The effect of competition for the floor on turn transition times has been investigated by comparing dyads interacting in friendly chats or arguments, with the latter resulting in shorter turn transitions (Trimboli and Walker, 1984), but not in multi-party compared to dyadic conversation.

To begin to fill this gap in the literature, we present an analysis of a corpus of unscripted casual conversations investigating the effect of group size (dyadic versus triadic) on turn transition times. The conversations were recorded in a laboratory, and the participants in the dyadic conversations also participated in the triadic conversations. We distinguished between two types of turn transitions, question-response sequences (QR transitions hereafter) and non-question-response turn transitions (non-QR transitions). The rationale for this is that questions often specify a specific respondent even in multi-party settings (e.g., Holler and Kendrick, 2015) or address several participants as one collective unit (Lerner, 1993). This may reduce the competitive element of turn-taking compared to non-QR transitions. In non-QR transitions, the current speaker may also select the next speaker, but speakers may more frequently self-select. Hence competitive effects should be seen more clearly in non-QR than in QR transitions. Note that in the present study we do not distinguish between competitive overlap (i.e., overlap that would be perceived as interruptive) and non-competitive overlap (Ferguson, 1977; Beattie, 1982; French and Local, 1983; Schegloff, 2000, 2001). This distinction is undoubtedly an important one for understanding the phenomenon of overlap and its management in interaction. However, the focus of the present study is on turn transition times, that is, a continuous measure of the extent of the gap or overlap that occurs between speakers. This measure is related to previous studies that have measured response times in conversational corpora (see above) which also did not consider different types of overlap. For comparability, we here apply the same general measure.

In sum, we propose that turn transition times in multi-party conversation may be reduced for two related reasons: First, there is a statistical reason; the fastest of several planned responses determines the recorded turn transition time. Second, the speakers’ behavior may change when they compete for the turn. Note that we are not proposing that competition for the floor is the only, or even the most important, cause of short turn transition times in multi-party conversation. We view it as one factor that may be influential but has so far received little attention in the psycholinguistic literature on conversation.

## Study 1: Simulations

The aim of the simulations was to examine how much the response time distribution obtained in a laboratory experiment would change if, instead of recording all responses, for each trial only the fastest response from two or more participants were recorded. The observed response times, on which the simulations were based, come from an experiment by Meyer et al. (2018, Experiment 1), which we briefly describe below.

### Experiment and Dataset

The participants were 21 adult native speakers of Dutch. The data from one participant were excluded due to technical failure. The participants were asked to listen to recorded questions about the objects on their screen (e.g., “Heb je een groene sweater?” English “Do you have a green sweater?”) and to respond as quickly as possible with “ja” (yes) or “nee” (no). There were always four objects on the screen. The referent object (the sweater in the example) was always included in this four-object display, but not necessarily in the color mentioned in the question. There were two experimental conditions, called the monochrome and the multi-color condition. In the monochrome condition all objects had the same color. In the multi-color condition they had different colors. This affected when the participants could begin to plan their response. For instance, when the question was about a green sweater and all objects on the screen were green, the participants could prepare to answer affirmatively as soon as they had comprehended the color adjective. Similarly, when all objects were white, the participants could begin to prepare a negative answer as soon as they had comprehended the adjective. In contrast, when the four objects on the screen had different colors, the participants could only begin to prepare a response after they had comprehended the noun as well. Thus, the Early Planning Hypothesis predicts that the response times should to be shorter in the monochrome than in the multi-color condition. The experiment included 256 trials, with 128 monochrome and 128 multi-colored displays. 64 monochrome and 64 multi-colored displays required an affirmative answer. The remaining displays required a negative answer.

A native Dutch speaker transcribed the utterances. Incorrect responses (less than 3% of the responses) and outliers, i.e., responses times deviating by more than 2.5 standard deviations (SD) from the condition mean (0–3% of the responses per condition) were excluded from the analyses. Table 1 shows the results for the remaining responses. As expected, participants were faster to respond in the monochrome condition than in the multi-color condition. In addition, affirmative answers were overall given faster than negative ones. The benefit for affirmative over negative answers was smaller in the monochrome than in the multi-color condition. For the statistical analysis and a discussion of the findings, please refer to the original paper.

**TABLE 1:** Means (with standard deviations [sd] in parentheses) and medians (with median absolute deviation [mad] in parentheses) observed in each condition of Experiment 1 in Meyer et al. (2018), simulated means and medians for pairs of participants, gain (difference between observed and simulated means and medians).

Display	Response	Observed responses		Simulated pairs (winners)		Gain	
		Mean (sd)	Median (mad)	Mean (sd)	Median (mad)	Mean	Median
Multi-color	Negative	417 ms (177)	325 ms (133)	325 ms (107)	276 ms (77)	92 ms	48 ms
Multi-color	Affirmative	344 ms (172)	245 ms (112)	252 ms (103)	217 ms (71)	92 ms	28 ms
Monochrome	Negative	200 ms (249)	160 ms (281)	72 ms (179)	85 ms (189)	128 ms	75 ms
Monochrome	Affirmative	234 ms (241)	200 ms (181)	96 ms (182)	134 ms (191)	149 ms	66 ms

### Methods

For the simulations, we created simulated pairs of participants, and for each simulated trial selected the fastest response time: First, we paired each of the 20 participants with each of the other participants, yielding 190 pairs. To obtain simulated data for each trial, response times were selected at random from each participant’s data per condition (for instance from the response times in the monochrome affirmative condition). Thus for each trial, two response times, one from each participant, were selected. This was done 32 times per condition, simulating the 32 trials of the experiment. For the entire experiment, this yielded a dataset of 44,442 response time pairs. The shorter of the response times was selected and plotted as the “winning” response time per trial.

### Results and Discussion

**FIGURE 1:**
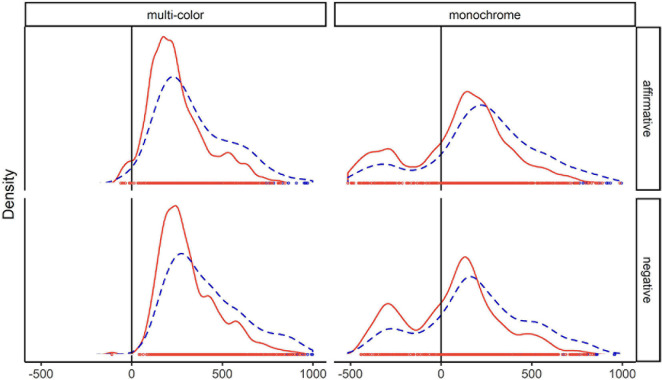
Density plots of all response times (blue) and “winning” times (orange) per condition. Data from Meyer et al. (2018, Experiment 1).

Figure 1 shows as density plots how the observed response times per condition (in blue) and the simulated “winning” response times (in orange) were distributed. As can be seen, the distributions of “winning” response times peaked earlier and had thinner right tails, i.e., included fewer long response times.

The simulated condition means and medians of the winning response times are tabulated in Table 1 next to the empirically observed means and medians for all responses. The two rightmost columns of the table show the gain, that is the differences between the means and median observed in the experiment and in the simulation.

As the numbers in the gain columns show, in the multi-color conditions, the estimated mean response time was shorter by 92 ms in both the affirmative and negative response condition for the “winning” responses compared to all responses. The medians changed less, by 28 and 48 ms for affirmative and negative responses, respectively. This is because medians are less affected by extreme values than means. In the comparison of the two response times per trial and the selection of the fastest one the longest response times are most likely to be discarded.

In the monochrome conditions, the response distributions were bimodal, as can be seen in the density plots. This is because here participants could, but did not have to, respond before the end of the question. As all objects on the screen had the same color, they could respond as soon as they had understood the adjective (“Do you have a green?”), which yielded response onsets shortly before the end of the question (“.sweater?”). However, participants often only responded after they had heard the entire question, yielding much later response onsets. The average reduction in mean response time was 149 ms for affirmative and 128 ms for negative responses. The medians were reduced by 66 and 75 ms, respectively.

**FIGURE 2:**
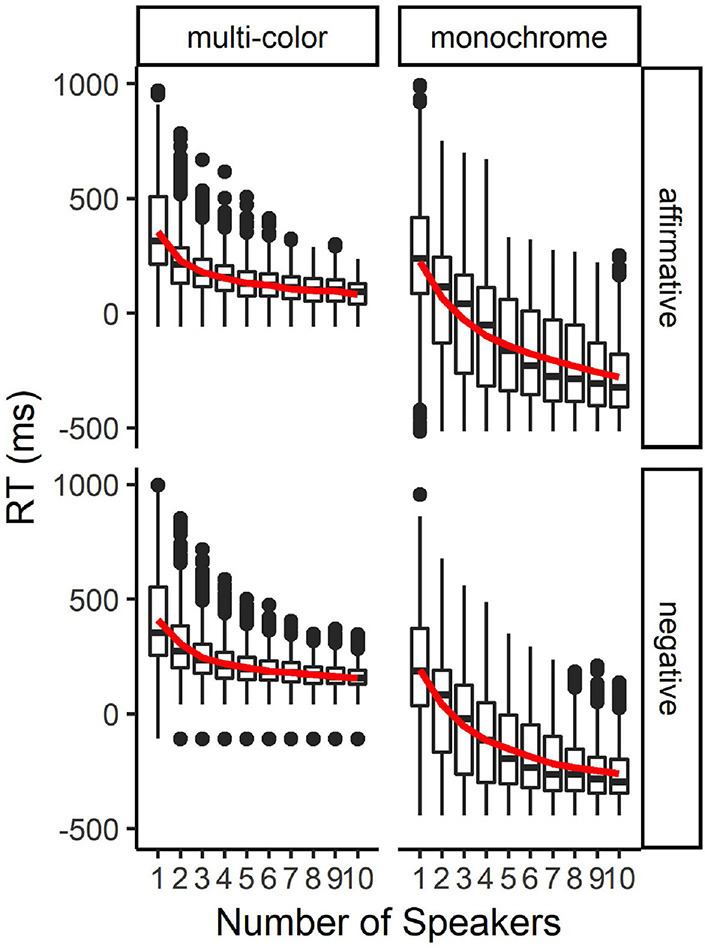
Box-whisker plots for the simulated reaction times for sets up to 10 participants. The boxes give an impression of the 25%, 50% (median), and 75% quantiles of the data. The lines show variability outside the 25 and 75% quantiles; outliers are plotted as individual dots. The red line gives an impression of the overall trend. It shows the change in mean response time with an increasing number of speakers.

In further simulations we assessed how the typical “winning” response times changed when responses from more than two participants were included and compared, and the fastest response time was selected. The simulations were run in the same way as described above, but instead of pairs, we generated sets of up to ten participants. Figure 2 shows how the mean of the “winning” response times changed with increasing group size. The mean response times first decreased substantially, but plateaued at a group size of five or six participants because the dataset included only a few valid response times below the plateau. In other words, since the task in, for instance, the multi-color condition could not reliably be completed correctly in less than 200 ms after the offset of the question, the number of data points entering the comparison did not reduce the mean “winning” response times below that plateau.

The goal of the simulations was to show how the recorded mean response times would change when, instead of including all responses, only the fastest, “winning,” response per trial from a sample of two or more participants was recorded. The key point–that the mean of the fastest response time is bound to be shorter than the average time of all response times–could have been made without the simulations. However, we hope that the simulations make the point more apparent for the reader. In addition, we obtained estimates of how much the mean and median response times across all responses versus the fastest responses differed from each other. Whether similar values would be obtained for other datasets remains to be seen. The differences should depend on the properties of the response distributions. When the participants already perform, on average, close to the human performance limit and when there are few slow responses due to, for instance, lapses of attention, the difference will be smaller than when the participants respond more slowly, and/or more erratically. However, regardless of the properties of the distributions, the mean of the fastest response times will always be lower than the mean of all response times.

We simulated the effect of selecting all responses versus a subset of the responses of the participants in an experiment. We did not simulate any changes in the behavior of the participants that might occur when they actually compete with each other. Whether and how competition affects participants’ response times in a laboratory setting can be assessed in further work. The point of the simulations was to illustrate the potential impact of a difference in the data from laboratory experiments on speech planning and corpora of conversational turn-taking, which often include multi-speaker conversations. The experimental data include all response times, but, as explained before, the corpus data typically only include the fastest responses of speakers competing for the floor. This may contribute to explaining why turn-taking in multi-party conversations appears to be so fast, compared to participants’ response times in laboratory experiments.

Note (Figure 2): Outliers deviating more than 2.5 SD from the condition mean were removed from the dataset. For the negative-multi-color condition, the lower limit was 112 ms. One outlier (−109 ms, one response from one of the participants) was not filtered out. As this is always the fastest response in any pair/set, this same outlier appears in every box plot.

## Study 2: Corpus Study

The aim of the corpus study was to examine how group size may influence turn transition times, i.e., the gaps and overlaps between turns in conversation. We expected turn transitions to be faster in triadic than in dyadic conversations. This should be the case for two reasons: First, in a triadic conversation two speakers may plan a response to a turn but only the fastest response enters the analysis and thus determines the turn transition time. As shown by the above simulations, the mean of these fastest turn transition times should be shorter than the mean of all turn transition times from responses provided by a single speaker in a dyadic conversation. Second, competition for the turn is more likely to arise in triadic than dyadic conversations. These influences should affect turn transition times in non-QR transitions, i.e., turn transitions that are not questions followed by responses. We did not expect a strong group size effect for question-response sequences (QR transitions), since here the current speaker often selects a next speaker, thus reducing competition for the floor.

### Materials and Methods

#### Participants and Corpus Creation

We analyzed a corpus of 12 dyadic and 12 triadic conversational interactions, each about 20 min in length. The conversations were selected from a larger corpus, based on the amount of pre-existing relevant annotation from a previous study (Holler and Kendrick, 2015). All conversations involved acquainted native speakers of English, with each participant forming part of one group only (i.e., there were 36 unique speakers in total, two thirds who took part in both the dyadic and the triadic conversation, and one third only in the triadic conversation). The participants’ ages ranged from 18 to 68 years (21 female).

Participants arrived in groups of three and were recorded while participating first in a 20-min triadic conversation followed by a 20-min dyadic conversation. To create the latter, one of the three people was asked to leave the recording laboratory. Throughout the session, the participants’ eye movements were recorded to address questions outside of the scope of this paper (e.g., Holler and Kendrick, 2015; Kendrick and Holler, 2017). Which participant was excluded from the second part of the session depended solely on the quality of the eye movement data acquired in the first session.

The conversations were unscripted and unprompted in terms of topic. Participants were left alone in the recording laboratory and asked to talk to each other as if they were engaging in casual conversation outside of the laboratory environment. They could talk about anything they liked, except for topics which might make their partners feel uncomfortable, for instance for ethical reasons. The entire test session per group lasted around 2 h, including set-up, instructions, two 20-min conversations, and obtaining a range of questionnaire measures at the end of the session, which were not used in the present analyses.

The recordings were made in a laboratory setting at the Max Planck Institute for Psycholinguistics in Nijmegen (Netherlands) and later, when recruitment of native English speakers in Nijmegen became too slow, at the University of Manchester (Manchester, United Kingdom). The audio recordings were made using uni-directional head-mounted microphones (Shure SM10A) which are suitable for detailed phonetic analyses of the audio signals from the individual speakers. For details on the laboratory set-up and equipment see Holler and Kendrick (2015). The two phases of data collection were approved by the Social Sciences Faculty Ethics Committee, Radboud University Nijmegen and the School of Psychological Sciences Ethics Committee, University of Manchester, respectively.

#### Measurement of Turn Transition Times

We distinguished turn transition times for question-response sequences (QR transitions) from other turn transitions (non-QR transitions). For the analysis of QR transitions, the full 20 min of each conversation (i.e., 480 min in total) were selected. To identify QR transitions we used the coding scheme developed by Stivers and Enfield (2010), which uses both formal criteria (e.g., syntactic marking) and functional criteria (i.e., the utterance seeks to elicit information). This resulted in 459 QR transitions for the dyads (mean = 38.25, SD = 18.85) and 497 for the triads (mean = 41.42, SD = 23.63).

For the analysis of non-QR transitions, the first 5 min of each conversation were selected (i.e., 120 min in total). All speaker changes were included, except for the following categories: (a) QR transitions; (b) backchannel responses, such as “mhm,” “uhu” (Yngve, 1970; also referred to as continuers by Schegloff, 1982, such utterances are usually considered as passing up the opportunity to take the turn), (c) turn transitions in which the first speaker ended their turn mid-speech due to being interrupted, and (d) turn transitions that involved extended silence, for instance because speakers were thinking of a new topic (e.g., Hoey, 2015); (e) transitions where the coder could not confidently identify first and next speaker because of too much overlapping talk; (f) transitions where the next turn started with laughter. This left 290 turn transitions for the dyads (per dyad mean = 24.17, SD = 11.42) and 259 turn transitions for the triads (per triad mean = 21.58, SD = 9.41) for the analyses.

Turn transitions were measured from the offset of vocalization of the first turn to the onset of vocalization of the next turn. This meant that turn transitions involving overlap resulted in negative numbers, and turn transitions involving a gap resulted in positive numbers. Vocalizations were any form of verbal utterance. Audible inbreaths, coughs, and such like were excluded (see also Kendrick and Torreira, 2015). The onsets and offsets of the turns were determined through inspection of their waveforms and spectrograms using Praat (Version 6.1; Boersma and Weenink, 2001). The corpus was annotated manually to select certain types of contributions and excluded others, such as to exclude backchannel responses (see above). These may look like speaker switches, but since they invite the current speaker to continue, they must be distinguished from actual turns. As a result, our analyses are based on a smaller corpus than may be achievable based on automatic coding of speaker contributions, but it allows us to draw clearer conclusions.

#### Statistical Analysis

We excluded as outlier turn transition times deviating by more than 1.5 times the interquartile range (i.e., >q0.75 + 1.5 × IQR or <q0.25-1.5 × IQR). This was done separately for the dyads and the triads, combining QR and non-QR transitions within those groups. There were 90 outliers (5.98% of the data), 67 QR transitions and 23 non-QR transitions. The data included in the analysis were characterized by a residuals distribution low in skewness and kurtosis (see OSF analysis script) with values falling well within the range of those acceptable for a normal distribution. We thus considered the assumptions underlying linear mixed effects models met. Means, medians, and modes have been calculated to give a comprehensive description of the data (modes were calculated based on Gaussian kernel density estimates, function “locmodes,” multimode package).

The data were analyzed using R (Version 4.0.4; R Core Team, 2020) and the lme4 package (Version 1.1.27; Bates et al., 2015). A linear mixed effects model was fitted to predict turn transition duration, with group size (coded using sum-to-zero contrasts with dyads coded as +0.5 and triads coded as −0.5) and transition type (also coded using sum-to-zero contrast, with QR coded as +0.5 and non-QR coded as −0.5) as fixed effects as well as their interaction. Model diagnostics showed that this model failed to predict negative turn transition durations for most groups, despite the presence of many negative turn transitions in all groups. Therefore, an additional term for “overlap” was added to the model to indicate whether a particular turn transition was positive or negative (see Table 2), resulting in a substantially improved model fit [χ^2^(7) = 1124.1, *p* < 0.001]. For the participant taking the turn, by-participant intercepts and slopes were included for transition type and overlap. As the model already showed signs of approximate singularity, no interaction between the by-participant slopes were included. The participant identifiers were distinct for dyads and triads. As such, including a by-participant slope for group size was not possible. However, the by-participant contribution was captured by distinct by-participant intercepts for each conversation. To calculate *p*-values, Satterthwaite’s method was used to compute the denominator degrees of freedom with the lmerTest package (Kuznetsova et al., 2017).

**TABLE 2:** Linear mixed model fit by maximum likelihood for the corpus data.

**Formula:** turn_transition_time ∼ 1 + overlap/(group_size × transition_type) + (1 + overlap + transition_type | T2_ID)					
**Model summary**					
AIC	BIC	logLik	deviance	df.resid	
19,857.3	19,936.1	−9,913.7	19,827.3	1,400	
**Scaled residuals**					
Min	1st Quartile	Median	3rd Quartile	Max	
−2.3786	−0.7638	−0.0828	0.6567	3.2764	

**Groups: Term**	**Variance**	**Std. Dev.**	**Corr**		

**Random effects**					
T2_ID: Intercept	5,834.8	76.39			
T2_ID: overlapTRUE	5,634.0	75.06	−0.99		
T2_ID: transition_type[S.QR]	170.7	13.06	0.99	−1.00	
Residual	68,824.8	262.34			

Number of obs		1,415			
Number of groups (T2_ID)		60			

	**Estimate**	**Std. error**	**d*f***	***t***	***p***

**Fixed effects**					
(Intercept)	365.232	13.826	58.941	26.417	<2e-16^∗∗∗^
overlapTRUE	-636.064	18.832	56.934	-33.776	<2e-16^∗∗∗^
overlapFALSE:group_size[S.dyadic]	54.509	27.652	58.941	1.971	0.0534
overlapTRUE:group_size[S.dyadic]	22.122	26.022	55.580	0.850	0.3989
overlapFALSE:transition_type[S.QR]	7.033	18.350	944.847	0.383	0.7016
overlapTRUE: transition_type[S.QR]	-5.106	25.920	879.721	-0.197	0.8439
overlapFALSE:group_size[S.dyadic]:transition_type[S.QR]	-59.155	36.700	944.847	-1.612	0.1073
overlapTRUE:group_size[S.dyadic]:transition_type[S.QR]	42.578	51.840	879.721	0.821	0.4117

*t-tests use Satterthwaite’s method. Group size and Transition Type are sum coded (±0.5); overlap is dummy coded and yielded a nested analysis of Group Size and Transition Type within each level of overlap. The main effect of overlap simply indicates that negative turn transition times were shorter than positive turn transition times (which is true by definition).*

A secondary analysis using a logistic mixed effects model (see Table 3) was conducted to see whether the probability of a negative turn transition duration (i.e., overlap) was influenced by transition type or group size. The structure of this model matched the linear mixed model for the transition duration, but overlap was used as the response instead of as a predictor in the fixed and random effects.

**TABLE 3:** Generalized linear mixed model fit by maximum likelihood (Laplace approximation) for the corpus data.

**Formula:** overlap ∼ 1 + group size × transition type + (1 + transition type | T2_ID)				
**Model summary**				
AIC	BIC	logLik	Deviance	df.resid
1,782.7	1,819.5	−884.4	1,768.7	1,408
**Scaled residuals**				
Min	1st Quartile	Median	3rd Quartile	Max
−1.0534	−0.7232	−0.5812	1.2107	1.9376

**Groups: Term**	**Variance**	**Std. Dev.**	**Corr**	

**Random effects:**				
T2_ID: Intercept	0.12422	0.3525		
T2_ID: transition_type [QR]	0.07033	0.2652	−1.00	

Number of obs		1415		
Number of groups (T2_ID)		60		

	**Estimate**	**Std. Err.**	***z***	***p***

**Fixed effects**				
(Intercept)	−0.74698	0.07852	−9.513	<2e-16***
group_size [S.dyadic]	−0.39172	0.15649	−2.503	0.0123*
transition_type [S.QR]	0.13720	0.12931	1.061	0.2887
group size [S.dyadic]:transition_type [S.QR]	0.55533	0.25734	2.158	0.0309*

*The response family was binomial with a logit link. Group Size and Transition Type are sum coded (±0.5).*

### Results and Discussion

**FIGURE 3:**
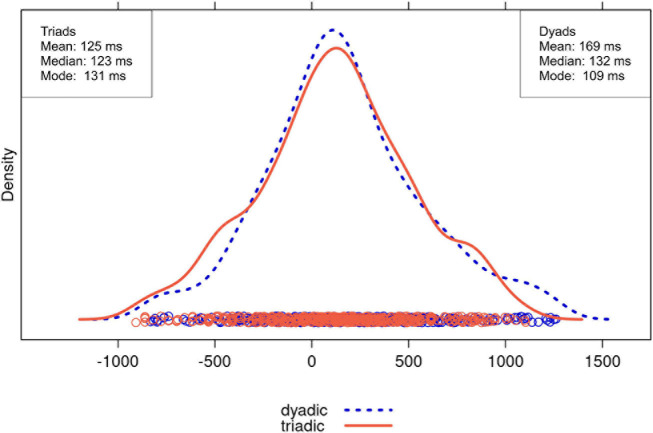
Distribution of turn transition times (in ms) for QR transitions for dyadic (dotted blue) and triadic (orange) conversations (outliers excluded).

**FIGURE 4:**
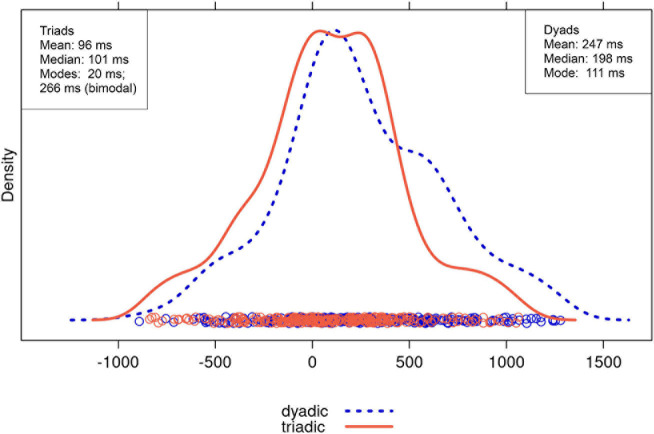
Distribution of turn transition times (in ms) in non-QR transitions for dyadic (dotted blue) and triadic (orange) conversations (outliers excluded).

We expected turn transitions to be faster in triads than in dyads, especially for non-QR transitions, that is, transitions that are not question-response sequences. Figures 3, 4 show the distributions of the transition times for QR and non-QR transitions, respectively, including the means and medians. These measures of central tendency, as well as the distributions displayed in the figures, all point toward the same pattern: dyads and triads did not seem to differ much in their turn timing for QR transitions, but triads had faster turn transition times than dyads for non-QR transitions, largely caused by more overlap (i.e., negative turn transition values).

More precisely, for QR transitions, the distributions strongly overlapped (Figure 3). The means for the two groups differed only by 44 ms, and the medians by 9 ms. Thus, turn transitions were not much faster in triads than dyads. The difference between the modes was even greater. The distributions for non-QR transitions also overlapped, but the distribution for triads was shifted leftward relative to the distribution for dyads (Figure 4). This indicates that relatively large negative turn transitions times (i.e., transitions with substantial speaker overlap) were more likely in triads than dyads. The means for triads and dyads differed by 151 ms, the medians by 97 ms, and the comparison of the dyad mode with the lower triad mode by 91 ms, all favoring the triads. The pattern looks different when considering the higher triad mode, indicating that fast responses were not characteristic for all of the triads’ non-QR transitions. However, the overall picture that emerges from the measures of central tendency is that group size markedly affected turn transition times for non-QR transitions, but not for QR transitions, as we expected.

The statistical models, reported in Tables 2, 3 confirmed this impression. The linear mixed effects model (Table 2) showed no significant effects of transition type or group size, but showed *some* evidence for faster transition times for triadic conversations *without* overlap [β = 54.5, *t*(58.9) = −1.97, *p* = 0.053]. That is, in those cases where turn transitions involved a gap, the gaps for triads were on average 55 ms shorter than those for dyads. Despite the model revealing no difference in terms of overlap duration for the two groups, we applied a second model to test whether group size nevertheless impacts on the *probability* of overlap. While overall (i.e., collapsing across group size), non-overlap was more probable than overlap (β = −0.75, *z* = −9.51, *p* < 0.001), this logistic model highlighted that the difference in mean turn transition duration between the groups was mainly driven by overlap being more probable for triads than dyads (β = −0.39, *z* = −2.5, *p* = 0.012). Additionally, there was an interaction between transition type and group size (β = 0.56, *z* = 2.16, *p* = 0.03). Decomposing this interaction with expected marginal means (using the R package emmeans, Lenth, 2021) showed that overlap was significantly more likely for triads than for dyads in non-QR transitions (β = −0.67, *z* = 3.3, *p* < 0.001) but not in QR transitions (β = −0.11, *z* = −0.56, *p* = 0.57).

We expected at most a small effect of group size for QR transitions because in such sequences the current speaker often selects the next speaker, which reduces competition for the floor compared to non-QR transitions. Questions in conversation can have many features that single out a particular addressee, such as the use of gaze or the addressee’s name (Sacks et al., 1974; Lerner, 1993, 2003). This holds for dyadic and multi-party conversation alike (see Holler and Kendrick, 2015). This means that QR transitions in multi-party conversation are often comparable to QR transitions in dyadic conversation. Bearing this in mind, it is not surprising that we did not observe reliably faster turn transitions for triads than dyads for QR transitions.

The absence of a group size effect for QR transitions might appear to be inconsistent with the results of the simulations reported above, which yielded strong group size effects for responses to questions. However, the simulations merely illustrated how means of response times change when they are based on all responses versus a subset of responses, specifically the fastest responses per trial from the set of participants. The linguistic properties of the responses used as the basis for the simulations, i.e., that they happened to be responses to questions, played no role for the outcome of the simulations. In other words, the simulations, while based on QR turn transitions, do not reflect the pragmatic attributes associated with such transitions and may, paradoxically, more accurately capture speakers behavior in non-QR transitions.

For non-QR transitions a substantial effect of group size was indeed obtained: The mean turn transition time for dyads was more than double the time observed for triads. The 151 ms difference corresponds roughly to the duration of a syllable in colloquial English (Greenberg et al., 2003). This difference arose at least in part, from a greater chance of speaker overlap in triads than in dyads. Thus, to the best of our knowledge the present results are the first to show that under comparable conditions, here conversations between acquaintances recorded in the laboratory, triads, on average, have shorter non-QR turn transitions than dyads.

Of course, this initial finding must be supported by further work. A strength of the current corpus is that the same participants were involved in the triadic and dyadic conversations. An obvious shortcoming is that for all speakers the triadic conversations preceded the dyadic ones. It is conceivable that the observed group size effect for non-QR transitions resulted from fatigue or decreasing motivation to talk in the second session of the study (i.e., the dyads). However, we think this is unlikely because fatigue or decreasing motivation should not selectively affect the non-QR transitions, but also the QR transitions, for which no group size effect was found. Nonetheless, further studies with counterbalanced order of group sizes, or between-participant designs, are needed to separate group size and order effects more conclusively. Moreover, it would also be good to consider larger corpora and corpora of different languages to see how generalizable the present effects are.

## General Discussion

We set out to investigate how the number of potential next speakers in a conversation may influence turn transition times. The starting point was the observation that in quantitative corpus analyses of multi-party conversation, only the fastest response to a turn enters the analysis. The reason is that participants who needed more time to respond may end up not producing a response, or they may produce it after someone else has taken the next turn, thus precluding it from the analysis. This may partly explain why turn transition times in natural conversations are so short compared to the response times in laboratory-based experimental studies of turn-taking. In the lab, participants are tested individually and all response times (except for some extremely long or short response times), feed into the mean. Indeed, the results of our simulation study confirmed that, up to a set size of about five participants, the more participants were entered into the model as potential responders, the shorter the mean “winning” response time became. This principle–that the mean of a subset of short response times will be lower than the mean of all response times in a dataset–is bound to play a role in multi-party conversation as well.

However, turn transition times may also be shorter in triads than dyads for other reasons, in particular differences in competition for the turn. At those points in conversation where turn transition becomes relevant (i.e., points of semantic, syntactic and pragmatic completion, Sacks et al., 1974; Ford et al., 1996), a current speaker either selects the next speaker or provides other participants with the opportunity to self-select. Since in multi-party conversation the “first starter acquires rights to a turn” (Sacks et al., 1974, p. 704), responding fast pays off. This does not apply to the results of the simulations where competitive behavior does not come into play. Thus, the strong group size effect, of 151 ms (for the means) seen for non-QR transitions was most likely due to a combination of sampling the fastest responses in the triadic corpus and increased competition for the floor.

The earlier corpus-based literature on turn transition times has concerned dyads, multi-party conversation, or a mixture of the two (e.g., Stivers et al., 2009; de Vos et al., 2015; Girard-Groeber, 2015; Holler et al., 2018). This complicates the comparison of turn transition times to response times obtained in laboratory settings from individually tested participants. We should note, however, that the inclusion of multi-party conversations in some of the corpora does not fully explain why the turn transition times are so much shorter than the response latencies in the laboratory. This is evident from the observation that turn transition times in dyadic conversation are also often shorter than response times of individual participants in the lab (e.g., Knudsen et al., 2020). Thus, there must be other factors at play that facilitate swift responding in natural conversations and/or hinder it in experimental settings. These factors could, for instance, pertain to the speakers’ motivation and engagement or to the linguistic structure and predictability of their utterances, as well as to their use of gaze, which has long been claimed to play an important role in turn-taking (Kendon, 1967).

While we have here focused on variables that may contribute to responses in conversation being faster than in the laboratory, there are also variables that occasion slow responses in conversation. These may relate to uncertainty about the content of the response (e.g., Fox and Thompson, 2010), or they may relate to pragmatics. For instance, dispreferred responses are often marked by longer turn transition times than preferred responses (Schegloff, 2007; Kendrick and Torreira, 2015) and initiations of repair (clarification requests resulting from trouble in understanding) generally occur after significant delays (Kendrick, 2015).

In sum, we propose that the absence of slow responses from typical corpus data, enhanced by the competition for turns, may partly explain the short turn transition times in everyday multi-party conversation. Further work is needed to substantiate this suggestion. In addition, the impact of other variables that may also influence turn timing (e.g., Roberts et al., 2015) remains to be identified. We think this would best be done by combining corpus analyses and experimental work, with the former providing fine-grained descriptive evidence about conversation in different settings and the latter uncovering the cognitive tools at the speakers’ disposition.

## Data Availability Statement

The datasets presented in this study can be found in an online repository: https://osf.io/um7ta/?view_only=9cd734fa2e9346e3aa89b3f958b46ef6.

## Ethics Statement

The studies involving human participants were reviewed and approved by Social Sciences Faculty Ethics Committee, Radboud University Nijmegen. The patients/participants provided their written informed consent to participate in this study.

## Author Contributions

JH and AM conceptualized and wrote the manuscript. PA designed the simulation. CD contributed to the design and data collection analyzed in Study 1. KK and JH collected the data for Study 2. KK and MG annotated the data for Study 2. PA analyzed the data for Studies 1 and 2. CD and MG contributed to the analysis for Studies 1 and 2, respectively. All authors contributed to the final version of the manuscript.

## Conflict of Interest

The authors declare that the research was conducted in the absence of any commercial or financial relationships that could be construed as a potential conflict of interest.

## Publisher’s Note

All claims expressed in this article are solely those of the authors and do not necessarily represent those of their affiliated organizations, or those of the publisher, the editors and the reviewers. Any product that may be evaluated in this article, or claim that may be made by its manufacturer, is not guaranteed or endorsed by the publisher.
